# 

**Published:** 1866-08

**Authors:** 


### Dental Register.

J. TAFT,

EDITOR AND PUBLISHER.

AUGUST, 1866.

MONTHLY:
THREE DOLLARS PER ANNUM, IN ADVANCE.

CINCINNATI:
WRIGHTSON & CO., Printers, 167 WALNUT STREET.

